# Osteoimmunology of Oral and Maxillofacial Diseases: Translational Applications Based on Biological Mechanisms

**DOI:** 10.3389/fimmu.2019.01664

**Published:** 2019-07-18

**Authors:** Carla Alvarez, Gustavo Monasterio, Franco Cavalla, Luis A. Córdova, Marcela Hernández, Dominique Heymann, Gustavo P. Garlet, Timo Sorsa, Pirjo Pärnänen, Hsi-Ming Lee, Lorne M. Golub, Rolando Vernal, Alpdogan Kantarci

**Affiliations:** ^1^Forsyth Institute, Cambridge, MA, United States; ^2^Periodontal Biology Laboratory, Faculty of Dentistry, Universidad de Chile, Santiago, Chile; ^3^Department of Conservative Dentistry, Faculty of Dentistry, Universidad de Chile, Santiago, Chile; ^4^Department of Oral and Maxillofacial Surgery, Faculty of Dentistry, San Jose's Hospital and Clínica Las Condes, Universidad de Chile, Santiago, Chile; ^5^INSERM, UMR 1232, LabCT, CRCINA, Institut de Cancérologie de l'Ouest, Université de Nantes, Université d'Angers, Saint-Herblain, France; ^6^Department of Biological Sciences, Bauru School of Dentistry, University of São Paulo, Bauru, Brazil; ^7^Department of Oral and Maxillofacial Diseases, University of Helsinki, Helsinki University Hospital, Helsinki, Finland; ^8^Department of Oral Diseases, Karolinska Institutet, Stockholm, Sweden; ^9^Department of Oral Biology and Pathology, School of Dental Medicine, Stony Brook University, Stony Brook, NY, United States; ^10^Dentistry Unit, Faculty of Health Sciences, Universidad Autónoma de Chile, Santiago, Chile

**Keywords:** osteoimmunology, oral, maxillofacial, periodontal disease, biomarkers

## Abstract

The maxillofacial skeleton is highly dynamic and requires a constant equilibrium between the bone resorption and bone formation. The field of osteoimmunology explores the interactions between bone metabolism and the immune response, providing a context to study the complex cellular and molecular networks involved in oro-maxillofacial osteolytic diseases. In this review, we present a framework for understanding the potential mechanisms underlying the immuno-pathobiology in etiologically-diverse diseases that affect the oral and maxillofacial region and share bone destruction as their common clinical outcome. These otherwise different pathologies share similar inflammatory pathways mediated by central cellular players, such as macrophages, T and B cells, that promote the differentiation and activation of osteoclasts, ineffective or insufficient bone apposition by osteoblasts, and the continuous production of osteoclastogenic signals by immune and local stromal cells. We also present the potential translational applications of this knowledge based on the biological mechanisms involved in the inflammation-induced bone destruction. Such applications can be the development of immune-based therapies that promote bone healing/regeneration, the identification of host-derived inflammatory/collagenolytic biomarkers as diagnostics tools, the assessment of links between oral and systemic diseases; and the characterization of genetic polymorphisms in immune or bone-related genes that will help diagnosis of susceptible individuals.

## Introduction

The maxillofacial skeletal structure has a complex geometry, adapted to the high mechanical requirements of the masticatory function. In this complex system, the alveolar bone accommodates the teeth and periodontal tissues while the basal bone provides support and insertion of masticatory muscles. Also, the mandible has two bilateral vertical rami that articulate with the base of the skull to form the temporomandibular joint (TMJ). Under physiological conditions, the bone undergoes continuous remodeling in a dynamic equilibrium of bone resorption by osteoclasts and bone formation by osteoblasts, at anatomically discrete sites known as basic multicellular units (BMUs) (1). Tight control of bone remodeling at the BMUs level is necessary to conserve structural integrity as the formation component needs to replace the exact amount removed by resorption. The strict synchronization of bone resorption and formation is referred to as coupling, a term that applies to each BMUs along with the skeleton (2). Bone coupling is controlled through a complex cellular communication network regulated by the signaling between osteoblasts, their mesenchymal pre-osteoblastic precursors, osteocytes, and osteoclasts and their monocytic precursors (1). Although circulating hormones, including PTH and 1,25-dihydroxyvitamin-D3, are considered to be the critical regulators of bone remodeling, it has become clear that locally generated cytokines are the key modulators of bone-cells communication and function (2).

The field of osteoimmunology has provided insight into the mechanics of osteoclast differentiation and activation during inflammation by immune cells and their soluble products. This process is fundamentally regulated by a triad of proteins of the tumor necrosis factor/tumor necrosis factor receptor family namely the receptor activator of nuclear factor κB ligand (RANKL), its functional receptor (RANK), and its soluble decoy receptor osteoprotegerin (OPG). Both soluble and membrane-bound RANKL can induce osteoclastogenesis through RANK in osteoclast precursors. Meanwhile, OPG inhibits the interaction between RANKL and RANK, and arrests osteoclastogenesis (3, 4). Under homeostatic conditions, RANKL is produced mainly by osteocytes, which have a higher capacity to support osteoclastogenesis than osteoblasts and are essentials for bone remodeling (5, 6). Interestingly, osteoblasts also produce RANKL which acts as an acceptor for vesicular RANK produced by mature osteoclasts. The osteoblastic RANKL-RANK cross-linking triggers RANKL reverse signaling, which promotes bone formation through the increased expression of early regulators of osteoblast differentiation (7). Under inflammatory conditions, however, the sources of RANKL are increased; immune cells such as particular subtypes of T cells and B cells can also produce RANKL (8). In addition, the local secretion of pro-inflammatory cytokines, such as IL-17 can induce the production of RANKL by osteoblasts and fibroblasts with osteoclastogenic capacity (9) suggesting a highly complex network of cellular origins of RANKL activity in tissues induced by inflammation.

In addition to osteoclast differentiation, inflammation impacts bone formation. Anabolic bone apposition is generally sustained by the expression of different growth factors such as fibroblast growth factors (FGFs), platelet-derived growth factors (PDGFs), insulin-like growth factors (IGFs), tumor growth factor β (TGF-β), bone morphogenetic proteins (BMPs), and Wnt proteins, released from the bone matrix or produced locally by different cell types (10–14). In particular, the Wnt/β-catenin and Bmp/Runx2 signaling pathways are essential for bone mass maintenance by regulating the differentiation and anabolic bone-formation activity of osteoblasts and osteocytes (15, 16). However, during inflammation, the activation of the classical (or canonical) NF-κB pathway inhibits the production of bone matrix proteins by decreasing the Bmp2-stimulated Runx2 and Wnt-stimulated β-catenin binding to osteocalcin and bone sialoprotein promoters (12). Inflammation also induces the production of the Wnt/β-catenin pathway inhibitors Dickkopf factor-1 (DKK1) and sclerotin (17, 18). The decline of inflammation restores the osteoblast functions by activating the canonical Wnt/β-catenin pathway (19), which has been shown to up-regulate OPG expression and inhibit osteoclast differentiation (20). Accumulating evidence, therefore, points to the failure of endogenous inflammation-resolution pathways as an underlying factor in the initiation and progression of chronic osteolytic inflammatory diseases and their relationship with systemic diseases.

In osteolytic inflammatory diseases such as periodontal disease, apical periodontitis, maxillofacial bone sarcomas and osteoarthritis of the TMJ, inflammation results in tissue destruction by the continuous release of osteoclastogenic mediators that counteract the production of bone-coupling signals. These otherwise different diseases share similar inflammatory pathways mediated by central cellular players, such as macrophages, T and B cells, that promote the differentiation and activation of osteoclasts, ineffective or insufficient bone apposition by osteoblasts, and the continuous production of osteoclastogenic signals by immune and local stromal cells. In the present review, we focused on the immune pathways that lead to the clinical signs of bone loss in different oro-maxillofacial diseases and possible translational applications of this knowledge.

## Periodontal Disease

### Definition and Pathogenesis of the Periodontal Disease

Among the osteolytic chronic inflammatory disorders of the jaws, periodontitis is the most well-defined and studied. The mechanisms underlying the pathogenesis of periodontitis are complex as periodontitis is a multifactorial disease that requires the combination of both a susceptible host and a dysbiotic polymicrobial community (21). Periodontitis is a significant public health problem due to its high prevalence, its cause of tooth loss, and its association with systemic diseases (22). Host susceptibility to periodontal diseases is the combination of genetic, epigenetic, behavioral, and environmental factors that modulate the immune response and the conditions of maintenance of the microbial community that colonizes the pathogenic biofilm (23, 24). The periodontitis-associated microbial communities not only stimulate but also exploit inflammation as a way to obtain nutrients for growth and persistence. The virulence factors of the bacteria can inhibit the antimicrobial functions and promote the pro-inflammatory and tissue-destructive properties of the host immune response to escape annihilation; consequently exacerbating and perpetuating the disease (21, 25). Thus, the interactions between the host and the microorganisms in periodontal disease are not only complex but also evolving.

### Cellular and the Molecular Immune Basis of the Periodontal Disease

The immune response during periodontitis involves different elements of innate and adaptive immunity. One of the first responders during the pathogenesis of periodontitis is the complement system, first recognized in early clinical studies that associated the disease with the presence of activated complement fragments in the gingival crevicular fluid (GCF) (26, 27). The use of animal models helped to clarify the role of complement-related mechanisms during periodontitis and alveolar bone loss (26, 28). These studies have highlighted the synergistic cross-talk of the complement with TLR pathways. For example, the activation of both C5aR1 and TLR2 by specific agonists resulted in the induction of significantly higher levels of pro-inflammatory cytokines in the gingiva (28). Mice lacking C5aR1 are resistant to bone-destructive diseases such as periodontitis and arthritis (29, 30), attributed to the critical role of the anaphylatoxin receptors in initiating neutrophils and macrophages adhesion and recruitment, necessary for the induction of bone resorption (31). Also, *P. gingivalis* targets C5aR to promote its adaptive fitness by manipulating the activation of TLR2 via the C5a-C5aR axis, allowing it to escape the IL-12p70-dependent immune clearance. This C5aR1-dependent evasion mechanism is crucial for the induction of microbial dysbiosis (29, 32).

Periodontal health is particularly sensitive to neutrophil functions and dysfunctions. Both hyper- and hypo-responsiveness of neutrophils have been associated with dysregulated inflammatory response and bone loss (33). Rare diseases related to defective extravasation of circulating neutrophils (LAD-1 deficiency) or neutrophil functionality (Papillon-Lefèvre syndrome) display a severe and fast-progressive form of periodontitis (34). If neutrophils cannot reach the gingiva, there is an overproduction of IL-23, IL-17, and G-CSF in the periodontium, attributed to macrophages, which in turn induces further inflammation and osteoclastogenesis (35). *P. gingivalis* possesses virulence factors that disrupt the neutrophil responses. For example, the LPS-induced TLR2 activation and cross-talk with C5aR inhibits the Myd88 but activates the Mal-PI3K pathway; this abolishes the antimicrobial response and PI3K-mediated phagocytosis while triggering the Mal-dependent inflammation (36). *P. gingivalis* can inhibit opsonization and phagocytosis, enhance neutrophil recruitment and respiratory burst, thus incrementing the neutrophil-associated inflammation and tissue damage (37). Neutrophils may also possess a hyper-inflammatory phenotype characterized by the over-expression of reactive oxygen species and pro-inflammatory cytokines (IL-1β, IL-6, IL-8, and TNF-α), which along with their other defective functions such as phagocytosis and chemotaxis, contribute to additional tissue-damage and comorbidity with other inflammatory diseases (38, 39). In the context of osteoimmunological regulation of periodontal diseases, neutrophils display heterotypic adhesion to osteoblast and modulate their function (40) and possess a regulatory role during microbial infection by secreting the anti-inflammatory cytokine IL-10 (41). Neutrophils acquire regulatory functions by direct cell-to-cell contact with regulatory T (Treg) cells or by exogenous IL-10 stimuli. The IL-10-producing neutrophils have been found in the purulent exudate collected from periodontal pockets in patients with chronic periodontitis; their role in the resolution of periodontal inflammation still needs to be investigated (42).

Even though macrophages are in low quantities in periodontal tissues (43), they participate in the pathogenesis of periodontitis as central players by initiating or resolving inflammation, contributing to tissue repair, activating lymphocyte-mediated adaptive immunity and mediating alveolar bone resorption and apposition (44). During inflammation, tissue-resident macrophages are expanded, and circulating monocytes are recruited to be differentiated into macrophage-like cells (45). Macrophages are divided into two functionally different subtypes: M1 classically-activated macrophages, produced in response to IFN-γ, TNF-α, IL-1β, and IL-6, with pro-inflammatory, antibacterial and antiviral functions; M2 alternatively-activated macrophages, produced in response to IL-4 and IL-13, with anti-inflammatory and tissue-repair/regeneration functions that expresses high levels of IL-10 (46–48). While these classes are clearly defined in mice; in humans, macrophages represent a continuum of highly plastic effector cells, resembling a spectrum of diverse phenotype states (47). Both M1 and M2 macrophages are increased in periodontitis compared to controls, yet the M1/M2 ratio is higher in periodontitis and is associated with increased expression of M1-related molecules such as IL-1β, IL-6 and matrix metalloproteinase (MMP)-9 (48, 49). Circulating monocytes/macrophages are affected by experimental periodontitis and display an M1 phenotype by overexpressing TNF-α and IL-6 (50). The temporal analysis of inflammation to healing osteolytic periodontal lesions showed a shift in the macrophage activation from inflammatory (CD80 and TNF-α expression) to resolving (CD206 expression) phenotype, which correlated to bone loss (51).

Lymphocytes are the majority of all CD45^+^ hematopoietic-origin cells within the normal gingival mucosa (43) and play a key role in osteoimmunology. The CD3^+^ T cell compartment is the dominant population in both health and disease, reflecting a 10-fold increase in total inflammatory cells (43). The analysis of alveolar bone resorption during *P. gingivalis*-induced experimental periodontitis in MHC-I or MHC-II deficient mice showed the destructive role for CD4^+^ T cells (52); yet effector-memory CD8^+^ T cells are present in normal gingival mucosa (43) suggesting a protective role for CD8^+^ T cells during periodontitis possibly due their ability to suppress osteoclastogenesis (53). Upon activation by the APCs, CD4^+^ T cells are polarized into distinct effector phenotypes depending on the nature of the antigen, co-stimulatory signals, and the local cytokine milieu (22). These phenotypes are Th1, Th2, Th9, Th17, Th22, and Treg, each with a particular transcription factor, often called a master switch, that modulates the phenotypic differentiation and particular effector-functions making these phenotypes highly plastic (54). Each phenotype has different involvement in the pathogenesis of periodontitis. They can be broadly classified in two axes: (1) Th1/Th17 pro-inflammatory and osteoclastogenic and (2) Th2/Treg mechanistically implied in the arrest of the disease and progression (55). Th9 and Th22, which are relatively new-subsets, have been scantily characterized in periodontal disease. Th22 cells were increased in gingival biopsies in periodontitis, associated with the increased osteoclastic activity, and triggered upon stimulation with the periodontal pathogen *Aggregatibacter actinomycetemcomitans* (56, 57).

Th1 cells produce pro-inflammatory cytokines such as IFN-γ, IL-12, IL-1β, and TNF-α, under the control of the transcription factor T-bet (22). The Th1-type of response, mediated by the production of IFN-γ, is necessary for both the control of microbial invasion and bone loss. The induction of periodontitis with *A. actinomycetemcomitans* in IFN-γ-deficient mice resulted in a less severe bone loss but impaired host defense against the microbial challenge, followed by a disseminated bacterial infection and mice death (58). *P. gingivalis* promotes the expression of type-1 interferons by disrupting innate immunological functions through degradation of Myd88, resulting in a constitutively priming of CD4^+^ T-cells by dendritic cells and leading to elevated IFN-γ and RANKL expression associated with increased alveolar bone loss. Blocking type-I IFN signaling prevented the destructive Th1 immune response and alveolar bone loss (59).

Th17 cells are the most osteoclastogenic type of T-cells, directly expressing and inducing RANKL expression on resident cells through IL-17 production, and necessary to sustain the host defense against the dysbiotic microbial community. Th17 cells produce IL-17A, IL-17F, and IL-22, under the control of the master switch RORγ, and the critical participation of the transcription factor STAT3 (60). In healthy individuals, Th17 cells naturally accumulate in the gingival mucosa with age and promote barrier defense. This growth depends on mechanical stimulation, such as chewing, which induces the production of IL-6 in epithelial cells, and is independent of commensal bacteria (61). The expansion of the Th17 cells during periodontitis, on the other hand, is dependent on microbial dysbiosis and requires both IL-6 and IL-23 production (62). These are predominantly resident memory Th17 cells, capable of quick responses and the primary producers of IL-17. A recent study in mice confirmed that IL-17 producing Th17 cells rather than γδT cells are involved in bone damage during periodontitis. IL-17 production is necessary for host defense against the invasion of oral bacteria (9). Also, a significant proportion of the IL-17 producing Th17 cells were exFoxp3Th17, cells that expressed high amounts of membrane-bound RANKL, suggesting that at some point, these cells might have had regulatory functions (9). Accordingly, patients with autosomal-dominant STAT3 deficiency (AD-HIES), are less susceptible to periodontitis (62).

The Th2 and Treg type of responses are implicated in the resolution of periodontitis (63). Th2 cells produce IL-4, IL-5, and IL-13, and mediate humoral immunity and mast cell activation in allergic reactions (22). Treg cells produce IL-10 and TGF-β and are crucial for the maintenance of immune homeostasis and tissue repair under the control of the master switch Foxp3 (64). Tregs inhibit osteoclast differentiation and their bone resorptive activity through the interaction of CTLA-4 with CD80/86 on osteoclasts and their precursors (29). Both Th2 and Treg cells express the chemokine receptor CCR4. The induction of periodontitis in CCR4^−/−^ mice presented a significant deficiency of Treg migration, associated with increased inflammatory alveolar bone loss (63).

B cells are practically not present in normal gingival mucosa (43), but they dramatically increase as the disease progresses, making it a distinct feature of the established periodontal lesion (22). During periodontitis, stromal cells and immune cells express different cytokines and chemokines such as IL-4, IL6, IL-5, CXCL13, and APRIL that induce B cell migration and support their survival in the periodontium (65, 66). Patients with periodontitis have a significantly higher percentage of CD19^+^CD27^+^CD382^−^ memory B cells and CD138^+^HLA-DR^low^ plasma cells while B1 cells, which have been previously described as a regulatory type of B cell (CD20^+^CD69^−^CD43^+^CD27^+^CD11b^+^) are decreased (67, 68). B cells/plasma cells are well-known for their humoral immunity. However, periodontitis progresses despite the presence of B cells and the induction of humoral responses against periodontal bacteria. The B cell-mediated IgG-dominant immune response might contribute to the pathogenesis of periodontitis (65, 68). Most infiltrating B cells present during periodontitis produce RANKL, suggesting that they can directly induce osteoclastogenesis (8). Indeed, the induction of experimental periodontitis in B cell-deficient mice showed significantly less bone loss (65). Latest data demonstrated the existence of a regulatory B cell subtype (Bregs) that can inhibit inflammation and support Treg differentiation through their production of IL-10. Bregs cells in humans have been identified as both CD19^+^CD24^hi^CD38^hi^CD1d^hi^ and CD19^+^CD24^hi^CD27^+^ cells (69). In mice, the functional IL-10-producing subset of Bregs, B10, have bone protective roles during periodontitis (70).

### Osteoimmunological Processes in the Periodontal Disease

The periodontium offers a unique environment to understand the interactions between the immune system and bone since it combines mucosal and skeletal tissues and the interaction between the host and the oral microbiota (21). The alveolar bone is susceptible to different types of mechanical stress and is continuously remodeled by the coupled action of osteoclasts and osteoblasts within the bone surface (3, 71). As reviewed above, the immune response caused by the dysbiotic microbiota during periodontitis dramatically enhances the production of local RANKL by different immune cell types such as Th17 and B cells. However, recent studies with specific cell type-depleted animals have highlighted the impact of RANKL production by osteocytes, osteoblasts, and periodontal ligament cells on osteoclast differentiation and alveolar bone loss (72). Osteocytes respond to inflammation, specifically to IL-6 and IL-17, by producing RANKL and increasing their osteoclastogenesis (73, 74). In a *P. gingivalis* and *Fusobacterium nucleatum-*induced periodontitis model, the genetic depletion of RANKL in osteocytes decreased the alveolar bone destruction and osteoclast differentiation (75). Osteocytes react to *P. gingivalis* LPS by producing sclerostin, which reduces osteoblastic bone formation by inhibiting the Wnt/β catenin signaling pathway (76).

**Figure 1:**
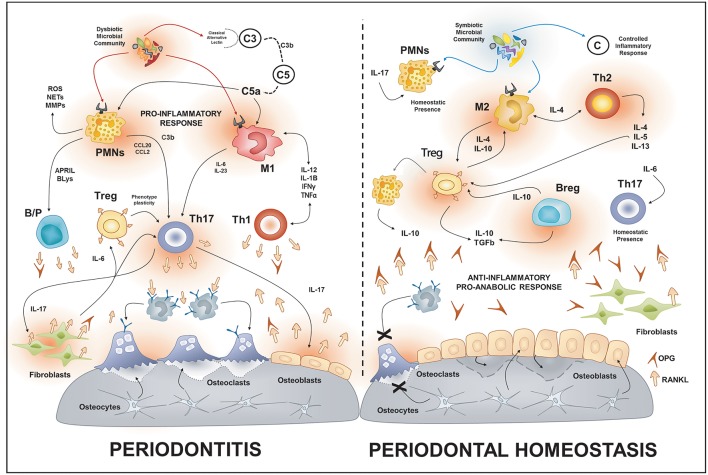
Osteoimmunology of periodontal disease. During periodontitis, the immune response induced by the dysbiotic microbiota enhances the production of local RANKL by different immune cells types such as Th1, Th17, B cells. Additionally, the increased gingival levels of IL-17 stimulate the membrane-bound expression of RANKL in osteoblasts and periodontal ligament fibroblasts; this provokes the activation of osteoclast and bone-loss. On the contrary, in health, different immune cells such as Tregs, Bregs, M2, and Th2 cells promote an anti-inflammatory and pro-anabolic state that sustain the alveolar-bone homeostasis. PMNs, polymorph nuclear neutrophil; B/P, B and plasma cell.

Osteoblasts and periodontal ligament cells also respond to IL-17, producing RANKL, and decreasing OPG production (77). The osteoblastic inflammatory-mediated RANKL production depends on the activation of the classical NF-κB pathway. The inhibition of NF-κB activation in osteoblastic lineage cells in mice reduces osteoclast numbers and RANKL expression induced by periodontal infection (72). Specific genetic depletion of RANKL production in osteoblasts and periodontal ligament cells during ligature-induced periodontitis reduces the alveolar bone resorption in an even greater extent that the RANKL depletion on CD4^+^ cells (9). Osteocytes, osteoblasts, and periodontal ligament cells significantly contribute to osteoclastogenesis during periodontitis by translating inflammatory signals into RANKL overexpression, often paired with OPG downregulation. This process results in the disruption of the coupling of bone resorption and apposition (Figure 1) (77). A recent study further demonstrated that bone matrix-derived products activate the NLRP3 inflammasome and stimulate osteoclast differentiation (78). The intracellular multi-protein complex known as the inflammasome functions as a molecular platform that triggers the activation of caspase-1, necessary to proteolytically process the biologically inactive form of IL-1β and IL-18 into mature cytokines. This conversion is key since RANKL acts in concert with TNF-α or IL-1β to regulate osteoclastogenesis (79).

### Translational Applications

Systematic approaches to control inflammation through immune modulation have been applied in different animal models of periodontal disease with the potential for translational applications. For instance, the blockade of the complement cascade at an earlier level, by the inhibition of C3 with AMY-101 (Cp40), is effective in a non-human primate model of periodontitis (80). This model is significantly more predictive of drug efficacy in a clinical setting since complement blockage inhibits inflammation in naturally occurring periodontitis (81). In murine models, the local delivery of CCL2 by control-delivered microparticles promoted the recruitment and differentiation of M2 macrophages, which in turn prevented alveolar bone loss (82). Similarly, the administration of CCL22-releasing microparticles prevented inflammatory bone loss by inducing the selective chemo-attraction of Treg in both murine and canine models of periodontitis (63). Rosiglitazone, a peroxisome proliferator-activated receptor (PPAR)-γ agonist, induced resolving macrophages with an M2-like phenotype that reduce bone resorption and enhance bone formation (51). The treatment with all-trans-retinoic acid or the synthetic retinoic acid receptor (RAR) agonist tamibarotene (Am80) improved the Th17/Treg balance and decreased the alveolar bone loss during periodontitis (83, 84). The treatment with RvD2 (a resolution agonist) prevented alveolar bone loss by inhibiting the systemic and gingival Th1-type of response in *P. gingivalis*-induced periodontitis (85). *In vivo* inhibition of Th17 differentiation by knocking *Stat3* or pharmaceutically inhibiting *Rorc* in CD4^+^ T cells led to significantly reduced alveolar-bone loss (up to 70%), reflecting their critical role in the induction of bone resorption by producing IL-17 and expressing RANKL (9). The antibody-mediated neutralization of APRIL or BLyS substantially diminished the number of infiltrating B-cells and reduced bone loss during the experimental periodontitis (65). The adoptive transfer of B10 cells, previously cultured with *P. gingivalis* LPS and cytosine-phospho-guanine (CpG) oligodeoxynucleotides, into mice with *P. gingivalis* and ligature-induced periodontitis, showed a significant reduction of bone loss and gingival inflammation, associated with increased local IL-10 production (70). Also, the gingival application of an optimized combination of CD40L, IL-21, anti-Tim1, which *in vitro* induces IL-10 production on B10 cells, inhibited bone loss in ligature-induced experimental periodontitis (86). Thus, the development of immune-based therapies has been proven effective in the prevention of bone destruction during experimental periodontitis *in vivo*. Current approaches to drug delivery and local applications of these therapeutic strategies in humans are being tested.

## Apical Periodontitis

### Definition and Pathogenesis of the Periapical Periodontitis

The infection of periodontal tissues in the periapical area following the bacterial invasion of pulp in the root canal system leads to the inflammatory destruction of the periodontal ligament, radicular cement and alveolar bone, which are the clinical hallmarks of apical periodontitis. Interestingly, the pathogenesis underlying the clinical presentation of apical periodontitis possesses outstanding parallels with that of periodontitis (87–89). Both conditions are initiated by an infectious stimulus and share pathological mechanisms of tissue destruction (chronic and exacerbated immune response that uncouples tissue balance) as well as the susceptibility traits and the treatment approach (eradication of the infecting microorganisms) (87, 90). Indeed, epidemiological data suggest that there is a correlation between the occurrence of apical periodontitis and marginal bone loss characteristic of periodontitis (91), reinforcing the existence of a common susceptibility profile.

### Cellular and Molecular Basis of the Periapical Periodontitis

Although microorganisms are essential for disease initiation, their presence is not sufficient to explain the pathologic phenomena that generate inflammatory destruction of the apical periodontium (92). The presence of a protected reservoir of microorganisms inside the root canal system precludes their eradication by immune defense mechanisms, generating a loop of constant activation and amplification of the immune response. Without active regulatory or suppressive signals, this amplification loop of the immune response results in a constant and exacerbated response, which causes the progressive destruction of periodontal support (87). This exacerbated immune response tampers with the normal turnover mechanisms of periodontal tissue, particularly bone, uncoupling bone formation from bone resorption leading to a net bone loss. Conversely, immunoregulatory mechanisms can provide a fine tune to immune effector mechanisms, resulting in a response that can control the spread of the infection outside the root canal system, while limiting the pathological tissue destruction. The balance shift toward deficient or excessive response can allow for infection spreading or uncontrolled periapical tissue resorption (93).

The treatment of apical periodontitis requires the disinfection of the root canal system and its obliteration with a biomaterial capable of maintaining a sterile environment and, in some refractory cases, the surgical elimination of periapical tissues (94, 95). The absence of reliable means to know in advance if the endodontic treatment will be successful or not is a critical weakness in endodontic therapy. Once the apical lesion has developed, there are no highly sensitive clinical or radiographic tools to predict if a lesion will acquire an activated phenotype -and continue to expand to the surrounding tissues- or will attain an inactive phenotype, resulting in the arrest of its progression or even remission and healing. Since not all subjects suffering from an infection of the root canal will develop apical periodontitis, it is logical to propose that a susceptibility profile is necessary for the occurrence of the disease The identification of putative genetic and molecular markers potentially responsible for the periapical immune-balance might help to discriminate susceptible or resistant subjects to improve the treatment outcome prediction (96). Additionally, periapical lesion development does not follow a linear pattern, alternating active and inactive phases, like the “bursts” progression model, described for periodontitis (97, 98). Therefore, the understanding of host response elements responsible for the switch from activity to inactivity can also contribute to elucidate the basis of susceptibility/resistance to lesions development.

From the genetic viewpoint, the overall hypothesis is that polymorphic variations in critical genes could contribute to increased risk to suffer from apical periodontitis. That possibility is investigated using a case-control approach, based on the comparison of a group of diseased subjects (i.e., cases) to an unaffected group of individuals (i.e., controls). However, in the context of periapical lesions, the use of a healthy population as control group disregards the classic case-control study definition, which states that a case-control study is designed to determine if exposure is associated with an outcome (99). The absence of the exposure (bacterial invasion of the pulp in the root canal system) disqualifies the healthy controls to be compared with susceptible individuals that develop periapical lesions upon the “exposure.” The study design, therefore, should comprise groups exposed to the same causal agent required for periapical lesions development, but with distinct clinical outcomes. The inclusion of theoretically resistant individuals (i.e., presenting deep caries without periapical lesions) have been found to improve the odds of identifying genetic factors that potentially contribute to increasing the risk to periapical lesions development (100, 101). A similar approach has been used in chronic periodontitis, with the use of chronic gingivitis subjects as a theoretically resistant population (99, 102).

Despite the inherent complexity to genetic association case-control studies, another useful approach to unravel the potential influence of genetic variants in apical periodontitis pathogenesis is to perform correlation analysis between the different genotypes/alleles and host response markers. For instance, MMP1-1607 polymorphism (rs1799750) is associated with increased expression of MMP-1 mRNA. MMPs are a family of collagenolytic enzymes responsible for the degradation and remodeling of the extracellular matrix. An increase in the expression or activation of MMPs without a parallel increase in their tissue inhibitors mediate numerous pathological processes, including apical periodontitis (103). In a sample of 326 subjects, the alternative variant of MMP-1 rs1799750 was associated with increased risk to suffer from apical periodontitis. Additionally, the cytokines TNFα, IL-21, IL-17A, and IFN-γ were associated with augmented transcriptional activity of MMP-1 in apical periodontitis, favoring its development (104).

Wnt/β-catenin signaling plays an essential role in bone biology, especially in the differentiation of osteoblasts and the suppression of bone resorption (105). The Wnt family in humans consists of 19 highly conserved genes that regulate gene expression, cell behavior, cell adhesion, and cell polarity (106). The polymorphic variations on the genes WNT3 and WNT3A were associated with increased susceptibility to apical periodontitis, specifically an intronic SNP in WNT3 (rs9890413) and a promoter SNP in WNT3A (rs1745420). The WNT3 (rs9890413) SNP is in an intronic region and to this date has no known function, so its putative mechanism of action to regulate apical periodontitis susceptibility is indefinite. On the other hand, a functional assays showed that the alternate allele G in the associated WNT3A (rs1745420), located in the gene promoter, increased promoter activity by 1.5-fold in comparison to the ancestral allele C. These findings suggested that this SNP may have a regulatory role in WNT3A expression and function (data not published).

The comparison of the gene expression signatures of periapical lesions with periapical tissues known to experience bone resorption or bone formation (i.e., in pressure and tension sides of teeth submitted to orthodontic forces) may allow for the discrimination of osteolytic activity status. Using this approach, it was possible to categorize apical granulomas in “active” or “inactive” according to their molecular profile of RANKL/OPG mRNA expression (107). Once the activity of the lesion was defined, it was possible to discriminate host response patterns associated with each subset. In this context, the inflammatory signature of 110 apical granulomas (persisting apical lesion after a technically adequate endodontic treatment, requiring surgery) and 26 healthy periapical tissues as controls were characterized (108). The apical granulomas were categorized as “active” or “inactive” according to the molecular profile of RANKL/OPG mRNA expression (107). The inflammatory signature was investigated by the expression of Th1, Th2, Th9, Th17, Th22, Thf, Tr1, and Tregs cytokines/markers. The cluster analysis revealed that “active” apical lesions were characterized by increased expression of TNF-α, IFN-γ, IL-17A, and IL-21, whereas “inactive” lesions expressed increased levels of IL-4, IL-9, IL-10, IL-22, and Foxp3. Interestingly, distinct patterns of IFNγ and IL-17 expression were described in periapical lesions. Lesions presenting a high RANKL/OPG ratio (active) overexpress IFNγ and IL-17 compared with inactive lesions. Additionally, active lesions can be clustered in groups presenting distinct patterns of IFNγ and IL-17 expression, suggesting that Th1/Th17 cytokines can drive apical periodontitis development independently (108). Accordingly, different studies describe that Th1 and Th17 responses can be mutually inhibitory (109). However, the Th1/Th17 interplay seems to be way more complicated than the mutually inhibitory activity, suggesting that collaborative and inhibitory phases may coexist in different disease stages. Also, cells presenting features of both Th17 and Th1 subsets, including Tbet and IFNγ expression, have been described in inflammatory and osteolytic conditions (110).

Therefore, the activity status of apical periodontitis may be determined by the relative enrichment of different Th subsets. Indeed, leukocytes subsets such as Th2, Tregs, and MSCs mediate a natural immunoregulatory response that suppresses apical periodontitis development. IL-4 (the prototypical Th2 cytokine) was described to induce the expression of CCL22, a main chemoattractant of Tregs (63). It is noteworthy that Tregs hallmark products, such as IL-10 and TGF-β, are described to boost MSCs immunosuppressive properties (111). Therefore, while all the details regarding the potential Th2, Tregs and MSCs cooperation remain to be unraveled, the existence of a protective/regulatory cellular network in inflamed periapical tissues seems feasible (89, 108).

### Impact of Osteoimmunology in the Periapical Disease

A recent study points to an unexpected potential trigger of a protective immunoregulatory response (112). While RANKL has a well-characterized role in the control of bone homeostasis, it can also play critical roles in the regulation of the immune system. Indeed, while anti-RANKL administration resulted in the arrest of periapical bone loss, it led to an unremitting pro-inflammatory response and impaired immunoregulation, restored by Tregs adoptive transfer (113). Therefore, RANKL seems to be responsible for trigger immunoregulatory feedback via Tregs induction, which in turn acts as suppressive elements (113). Notably, RANKL seems to play a fundamental role in linking the immune system with bone metabolism. Infiltrating immune cells are an important source of RANKL, but also resident bone osteoblast produce and secrete RANKL, most of the time under the regulatory influence of osteocytes following mechanical or endocrine stimulus (73, 75).

Despite the lymphocyte-centered paradigm of the most studies into apical periodontitis pathogenesis in the last decade, other leukocyte subsets (such as granulocytes) also can play significant roles in periapical lesion pathogenesis. For example, we determined that CXCL12 levels increase significantly in apical periodontitis. CXC ligand 12 (CXCL12 a.k.a. SDF-1) is a pleiotropic chemokine that regulates the influx of leukocytes to inflamed sites. In apical periodontitis, CXCL12 proved to be the primary molecular signal responsible for the recruitment of mast cells into the periapical inflammatory infiltrate during lesion development (114). This CXCL12/mast cell axis is significant as mast cells can secrete a variety of molecular signals and regulate many diseases (115, 116).

Another major cellular player during the first stages of the immune response in the pathogenesis of apical periodontitis is the neutrophil. These cells invade the apical periodontium in vast numbers and are in the front line of contention of bacterial infection. Despite being mainly associated with the direct killing of bacteria and tissue necrosis, neutrophils are also capable of releasing molecular mediators into the extracellular compartment and influencing the later stages of the response (117, 118). Accordingly, a significant increase in heat shock protein 27 (HSP27) and Serpin Family B member 1 (SERPINB1) protein levels were identified in apical periodontitis compared to healthy tissues (119). HSP27 belongs to the heat shock protein gene family and has an essential role in the inhibition of apoptosis in thermal and chemical stress, protecting the cells from injury in hostile environments (120). SERPINB1 is a potent inhibitor of neutrophil serine proteases and plays crucial roles in protecting PMN and other cells from apoptosis (121). The role of another Serpin family member (SERPINE1) has been demonstrated in the stabilization of apical lesions (122), thus pointing to a molecular pathway of Serpin family proteins regulating PMN functions and periodontal destruction in apical periodontitis. Importantly, this increased expression of HSP27 and SERPINB1 was compartmentalized to epithelial cells and infiltrating neutrophils in the inflammatory front (119). The expression of HSP27 and SERPINB1 was inversely correlated with markers of acute inflammation and markedly increased in apical lesion characterized as “stable/inactive.” This evidence suggests that HSP27 and SERPINB1 could be putative markers of lesion regression and useful to follow the outcome of endodontic treatment

Ultimately, most osteoclastogenic signals are controlled by osteocytes, whether directly by secreting osteoclastogenic signals or indirectly by entering apoptosis (123–125). The inflammatory milieu characteristic of apical periodontitis creates the necessary environment to favor pro-osteoclastogenic signaling and net bone loss (72). The communication network established between osteocytes and osteoblast is capable of sensing delicate environmental changes and react to favoring the bone formation and resorption (126). The exacerbated and unrelenting immune response characteristic of apical periodontitis provides plenty of pro-resorptive signals that tilt the balance in favor of bone resorption (127, 128).

**Figure 2:**
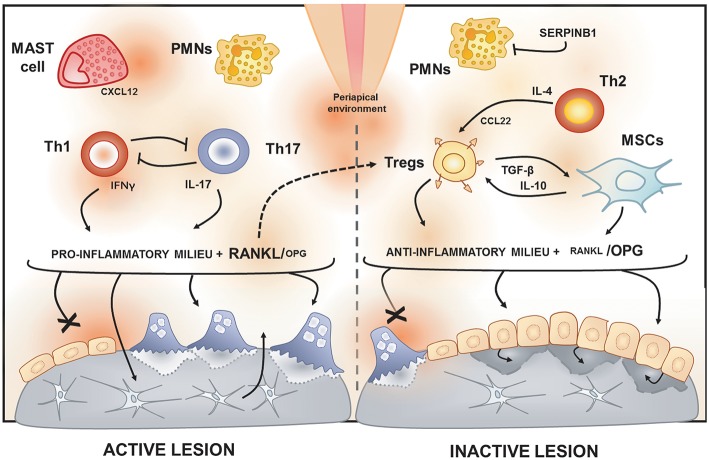
Immunological features of active and inactive periapical lesions. In the periapical environment, the presence of endodontic pathogens and its products (such as LPS) triggers the host inflammatory immune response. The analysis of gene expression signatures of periapical lesions allows for the discrimination of osteolytic activity status in “active” or “inactive” according to RANKL/OPG expression ratio. Active periapical lesions are characterized by high RANKL/OPG ratio is an association with a pro-inflammatory milieu, which includes a high level of IFN-g and IL-17. Distinct patterns of IFNg and IL-17 expression were described in periapical lesions, suggesting that Th1 and Th17 subsets, described to be mutually inhibitory, can drive apical periodontitis development independently. The presence of PMNs and mast cells also have been associated with lesion activity. Conversely, inactive lesions are characterized by a low RANKL/OPG ratio is an association with an anti-inflammatory milieu, which is supposed to involve a cooperative immunoregulatory network composed by Th2 and Tregs subsets, as well by MSCs. The prototypical Th2 cytokine IL-4 is described to induce the expression of CCL22, a main chemoattractant of Tregs. Noteworthy, Tregs hallmark products, such as IL-10 and TGF-β, are described to boost MSCs immunosuppressive properties. Interestingly, a recent study points to RANKL as an unexpected immunoregulatory feedback trigger via Tregs induction.

Taken together, recent findings point to a complex and multilevel regulatory network that underlies the clinical presentation of apical periodontitis (Figure 2). Infecting agents and immune defense mechanisms are in opposing trenches in an all-out war leading to apical lesion formation or regression. As in many other diseases characterized by inflammatory tissue destruction, the regulatory features of the immune system have a disproportionally important role in the progress and outcome of the disease. Despite extensive efforts, there is still much to be investigated and learned before we can develop a comprehensive molecular model capable of guiding changes in clinical conducts leading to improved clinical outcomes in the treatment and management of apical periodontitis.

## Biologically-Based Diagnostics and Therapeutics to Manage Oral and Systemic Health in Periodontitis Patients

Marginal periodontitis and periapical periodontitis are the most common oral diseases involving alveolar bone loss (129). Substantial research supports the positive association between periodontitis and several systemic diseases such as cardiovascular diseases and diabetes, while growing evidence is unveiling an analog connection with periapical lesions. Recently, periodontitis has also been associated with the onset and development of oral and extraoral cancers, and their fatal outcome (130–132).

The result of periodontitis forms is a prolonged release of both host-derived inflammatory/collagenolytic mediators (e.g., arachidonic acid metabolites, cytokines, nitric oxide [NO], reactive oxygen species [ROS] and MMPs), and virulence factors generated by the dysbiotic perio-pathogens and/or endodontic pathogens in the etiologic microbial biofilm. Though this response is intended to restrain dissemination, this toxic “brew” impairs the host's immune response. The proposed mechanisms linking periodontitis and extraoral diseases involve the spread of bacteria from the oral cavity causing damage to other organs, the increase in inflammatory systemic burden, or an autoimmune response triggered by oral bacterial species (133–138).

Albeit clinical and radiographic examinations are the gold standard for the diagnosis of periodontal and periapical diseases, variations in the inflammatory profile might impact disease susceptibility and severity at both local and systemic levels (129, 139). Oral fluids (gingival crevicular fluid/GCF, mouth rinse, and saliva samples) obtained non-invasively from the oral cavity are critical sources for factors and/or biomarkers related to the metabolic activity of periodontal tissues. Quantitative point of care (PoC)/chair-side technologies are emerging as available tools to monitor periodontal conditions, including orthodontic tooth movement, periodontitis, apical periodontitis, and peri-implantitis, whereas key inflammatory mediators can be targeted for therapeutic purposes. Complementary salivary/ oral fluid MMP-8 determinations aid to identify periodontal loss and inflammation in line with clinically deepened periodontal pockets, bleeding on probing, and radiographic alveolar bone loss (140–144). MMP-8, MMP-9, TRAP-5, and MPO demonstrates very high diagnostic accuracy in GCF for discriminating periodontitis, apical periodontitis, gingivitis and/or healthy periodontium, supporting their usefulness for PoC diagnostics (129, 142). Of interest, oral fluid biomarker analysis has shown usefulness in extraoral conditions or diseases (141, 142), whereas GCF placental and inflammatory markers proved the diagnostic potential for preeclampsia (145) and gestational diabetes mellitus in pre-symptomatic women (146), revealing new emerging spectra for oral fluid applications.

Up to now, several studies have explored the associations between tooth loss, oral infections, CVD and diabetes, and it is widely accepted that low-grade systemic inflammation, as measured by CRP and other biomarkers, influences their development and progression. Currently, there is substantial evidence supporting that marginal periodontitis imparts increased risk for future atherosclerotic cardiovascular disease, in which the exacerbated inflammatory burden favors atheroma formation, maturation, and exacerbation. Periodontal treatment can reduce systemic inflammation as evidenced by a reduction in C-reactive protein (CRP), reduce the levels of oral fluid and systemic (serum) proinflammatory biomarkers of tissue destruction and improve endothelial function and subclinical atherosclerosis, but the evidence is not yet conclusive (131, 147). Periodontitis also associates with elevated risk for dysglycaemia and insulin resistance, as well as incident type 2 diabetes, whereas the latter is also an essential modifying factor for periodontitis. Evidence supports that periodontal therapy seems to improve glycemic control, although studies involving long-term follow-up are also inconclusive (133). Despite the associations between endodontic infections, CVD and diabetes have not been thoroughly explored; emerging evidence sustains an analogous link (134).

Most available mechanistic studies seeking for an association between apical periodontitis and the systemic inflammatory burden lacks adequate control for confounders. Often, a clinically heterogeneous mixture of acute and chronic forms of apical periodontitis is included, and participants are older than the main risk group, resulting in overall inconclusive evidence for hsCRP. Few recent studies accounting for these variables reported early endothelial dysfunction and up-regulation of pro-inflammatory cytokines, including IL-1, IL-2, IL-6, reactive oxygen species, as well as asymmetrical dimethylarginine in serum from young adults with CAP compared to healthy volunteers (148). Recently our group demonstrated an association between apical lesions and cardiovascular risk based on CRP serum levels concentrations (135), but the systemic effects of endodontic treatment are yet unknown (134).

Recently periodontitis was proposed as an independent risk factor for cancer development, such as digestive tract cancer, pancreatic, lung, prostate, breast, uteri, lymphoma, and hematological cancer. Moreover, in population-based studies, periodontitis was strongly linked to cancer mortality, especially in patients with pancreatic cancer (130, 149, 150). Studies demonstrate a role of microorganisms such as Human Papillomavirus (HPV), Epstein-Barr virus (EBV) and *P. gingivalis* that could be detected in inflamed periodontal tissues and might favor cancer initiation at the oral cavity or distant tissues (151–153). Recently, *Treponema denticola*, a virulent proteolytic periodontopathogen, was found to promote the onset of oro-digestive cancers (154, 155). Besides oral pathogens, the local and systemic inflammatory responses associated with periodontitis can represent an indirect mechanism that could promote cancer development (130).

**Figure 3:**
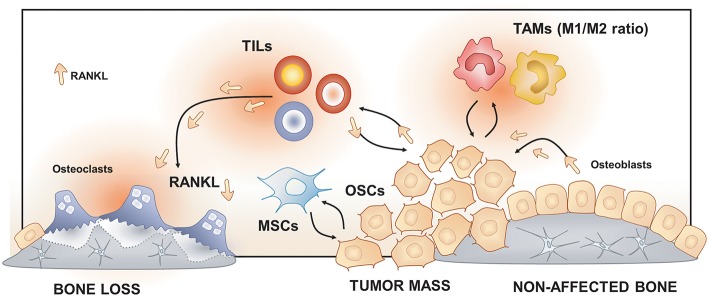
Osteoimmunology at the osteosarcoma (OS) niche. Tumor-Associated Macrophages (TAMs), Tumor-Infiltrating Lymphocytes (TILs) and Mesenchymal Stem cells (MSCs) regulate osteosarcoma cells (OSCs) proliferation, tumor mass progression, and metastasis. The RANK/RANKL signaling leads to both OSCs progression and increased osteoclast activation and bone loss in the OS niche.

Periodontitis derived-systemic low-grade inflammation can also be readily monitored in serum samples, by measuring acute-phase proteins such as C-reactive protein, to further diagnostically assess the risk. hsCRP measurement is especially recommended in subjects at intermediate cardiovascular risk to determine the need for treatment (156); hsCRP levels are also used to evaluate the success of an intervention such as the metalloproteinase inhibition with sub-antimicrobial doses of doxycycline to prevent acute coronary syndromes (MIDAS) (157, 158). In this way, biomarker's utility becomes clinically practical with the current availability of PoC chair-side biomarker analysis of oral fluids & serum, and host-modulation therapies such as non-antimicrobial doxycycline medications (Periostat®, now generic; and Oracea®) as pleiotropic MMP-inhibitors, and others such as omega-3 fatty acid derivatives (e.g., docosahexaenoic acid), i.e., the resolvins (Figure 3) (159–161). Thus, recently-developed strategies of personalizing the use of “host-modulation therapy,” when indicated by modern PoC chair-side diagnostic tests (140, 144), may significantly enhance the beneficial outcome of both the commonly-used oral therapy (scaling & root planing, and oral hygiene instruction) and its impact on the overall medical health of the patient. Further evidence of the need for modern, biologically-based diagnostics and therapeutics to manage the oral/systemic health of the patient is continuously emerging. These findings reinforce the view that modern, biologically-based oral-systemic health management requires a “two-pronged strategy” including diagnostic monitoring in oral fluids biomarkers, and optimally therapeutic suppressing these mediators with “host-modulation therapy” combined with microbial biofilm management. Both strategies are currently available to the dental clinician as the result of long-term and substantial basic and translational research.

## Bone Sarcomas (BS) Affecting the Maxillofacial Region

### Definition and Epidemiological Impact of Bone Sarcomas

Bone Sarcomas (BS) are rare primary mesenchymal bone tumors (<0.2% of malignant tumors of EUROCARE database), including osteosarcoma (OS), Ewing sarcoma (ES) and chondrosarcoma (CS) (162). BS affects more frequently the appendicular skeleton (lower limbs) than the craniomaxillofacial skeleton. In this area, maxilla and mandible are more affected bones over cranial bones (163). Thus, maxillofacial (MF) OS (MFOS), ES (MFES), and CS (MFCS) are considered malignant tumors of the maxillofacial region according to the 4th edition of the World Health Organization Classification of head and neck tumors (164). Here, we will focus specifically on MFOS, MFES, and MFCS, highlighting the role of immune response on their pathophysiology and, also, revising experimental approaches for therapy.

#### Maxillofacial Osteosarcoma (MFOS)

MFOS represent <10% of the total OS (165). Compared with the appendicular OS, which peaks in the 2nd and sixth decade, MFOS peaks in the 3rd decade (162, 163, 165). Typically, MFOS arise from the cancellous compartment rather than bony surfaces. MFOS affects the alveolar ridge of the mandible and posterior area of the maxilla (163, 166). MFOS can be categorized according to the predominant matrix as an osteoblastic, fibroblastic, chondroblastic, telangiectatic, or osteoclastic type (163, 165). At the X-ray imaging, MFOS appear as either as the osteolytic form with undefined margins or, the osteoblastic form, showing a sclerotic and sunburst structure caused by radiated bone spiculae (163).

#### Maxillofacial Ewing Sarcoma (MFES)

MFES is 1–4% of all ES which peak in both the first and second decades mainly in white Caucasian people, affecting equally both sexes (163). MFES is an aggressive, hemorrhagic, and rapidly metastatic malignant tumor affecting naso-orbital bones. MFES are characterized by an irregular lesion combining sclerosis and lucent zones compromising cortical bone. MFES also show the characteristic onion peel appearance and sunburst new bone formation, which correspond to periosteal osteogenic reaction (163).

#### Maxillofacial Chondrosarcoma (MFCS)

It accounts for 2% of all CS with a peak incidence during the 4th to fifth decades with a male predilection (ratio 2.4:1). It affects mostly the skull base, maxilla, and less frequent the orbit and, cartilage of the nasal septum. MFCS can be observed after malignant and benign diseases such as OS, fibrosarcoma, Paget disease, and fibrous dysplasia (163, 167). Histologically, chondrosarcoma of the craniofacial region can be divided into (a) the conventional subtype with myxoid and/or hyaline components, the most common form, is slow growing, and rarely metastatic; (b) the aggressive mesenchymal and dedifferentiated subtype, more aggressive and tends to metastasize and; the clear cell subtype, extremely rare (163). High-grade CS can induce metastasis, but local recurrence of curettage is a common feature of such tumors. Since the vast majority of literature describing the pathophysiology of BS focus on the non-maxillofacial OS, we will base our work upon these recent findings.

These three entities result from the disruption of differentiation of Mesenchymal Stem Cells (MSCs) into bone and cartilage cell lineages (168). In bone homeostasis, MSCs differentiate into stromal cells, which will contribute to both the hematopoietic and the skeletal niches (168, 169). At the skeletal niche level, stromal cells will activate transcription factors such as Runx2 to follow the osteogenic path to become functional osteoblasts or, Sox9 to follow the chondrogenic pathway to become cartilage cells (168) (Figure 3).

BS results from the interaction of both OS cells (OSCs), cancer stem cells, and their niche. OSCs are the malignant counterpart of osteoblasts. OSCs are mesenchymal-derived cells subjected to an initial oncogenic event altering the commitment from a mesenchymal cell toward an osteoblast by a mutation (e.g., *p53* and *Rb*) and/or aberrant Hedgehog and Notch signaling (168, 170). Within the tumor mass, cells exhibit high heterogeneous profiling that can be partly explained by the presence of cancer stem-like cells (CSCs), the clonal evolution of OSCs and the high heterogeneity of the local tumor microenvironment (168, 170). These cells are characterized by its self-renewing property, and they are proposed as responsible for tumor progression, resistance to chemotherapy, and initiate metastasis (168, 171). Based on the “seed and soil' theory of Paget, it is now well-recognized that OS, like other cancers, requires an adequate local microenvironment for its development (168, 171). This specialized microenvironment provides all metabolites and regulates the self-renewal process of CSCs (171). Interactions between OSCs, CSCs, and its niche may determine OS progression or dormancy and, potential drug resistance (171, 172).

### Cellular and the Molecular Immune Basis of Bone Sarcomas

Some clinical studies correlate survival rates of OS patients with both immune markers and the immune cell (lymphocyte/macrophage) ratio (173, 174). However, the role of the immune system in OS development remains still misunderstood. The relationship among OS niche and immune response may be explained by the fact that OSCs (and also, bone cells) are surrounded by bone marrow cells occupying the same bone marrow space. Within this space, hematopoietic precursors give rise to the immune cell population, lymphoid, myeloid cells, and mast cells. These cells will regulate both innate and acquired immune responses (173). Consistently; an immune infiltrates composed by monocyte/macrophages/dendritic cells and T-lymphocytes have been identified in OS tissues (173, 174). Although B-lymphocyte and mast cells are less represented in the OS tumor mass, they are far more distributed in the interface bone-tumor. Both lymphocytes and mast cells are essential sources of RANKL, becoming key players in the activation of osteoclasts, and then contributing to the osteolytic feature of OS (173, 174).

Macrophages are essential cells participating in bone homeostasis (173, 174). Macrophages, located in the vicinity of the tumor, are known as Tumor-Associated Macrophages (TAMs) (168). TAMs control local immunity, angiogenesis, and regulate tumor cell migration and invasion (168). Also, TAMs participate in the seating of cancer cells at the metastatic site by modeling the permissiveness of the host-tissues (168). TAMs are composed by a large variety of subpopulations which have been classified initially in M1 and M2 subtypes according to their differentiation and activities. M1, the pro-inflammatory macrophage subset, are classified as anti-tumor cells and associated with excellent survival rates, and M2, the anti-inflammatory macrophage subset, as pro-tumor regulators (168, 173, 174). Thus, in OS patients, a TAM-M1 predominant ratio over TAM-M2 was associated with better survival rates and the opposite, with poor prognosis (168, 173, 174). These associations may be explained by an immunosuppressive effect on intra-tumor T-lymphocytes, and pro-angiogenic effect exerted by TAM-M2 observed both preclinical models of OS and metastatic patients (168, 173, 174).

T-infiltrating Lymphocytes (TILs) are the second more prevalent infiltrated cell type in OS tissues and OS metastasis (174). Studies showed that selected subpopulations of T-cells (CD8^+^/FOXP3^+^) exhibit high reactivity again tumor cells compared with non-infiltrating lymphocytes (175). Thus, OS patients with elevated CD8^+^/FOXP3^+^ -ratio had better survival rates confirming the immunosuppressive role of TIL in OS pathogenesis (175, 176). TILs have higher cytotoxic properties again OS cells compared with circulating T-cells; however, OSCs secrete immunosuppressive molecules preventing the activation of TILs on the tumor site (175).

Beside immune cells, Mesenchymal Stem Cells (MSCs) have been reported as an essential regulator of OS behavior (177). Indeed, both bone and the bone marrow niche are rich in MSCs that are closely located to OS cells (178). Several studies demonstrated that MSCs establish active crosstalk with OSCs controlling OS progression and/metastasis (178). MSCs and early developed pre-osteoblasts communicate with OSCs by secreted vesicles containing mRNA, proteins, and miRNA modulating OSCs proliferation and stemness (e.g., ability to form sarcospheres, expression of stem-associated genes) (179). Reciprocally, OSCs are able to educate MSCs by tumor-secreted extracellular vesicles turning MSCs into OS extracellular vesicle-educated MSCs. These cells promote tumor progression and/or metastasis via secretion of IL-6, TGF-β and IFN-γ and by inhibiting T, B and NK cell proliferation (173, 174).

### Impact of Osteoimmunology in Bone Sarcomas

Bone remodeling is controlled by osteoblasts and osteoclasts, which are responsible for bone formation and resorption, respectively. Bone remodeling is regulated by the RANKL/RANK/OPG triad (180). RANKL expressed as a membranous or secreted form by stromal and osteoblastic cells which binds to RANK, a transmembranous receptor expressed by pre-osteoclast. Their interaction leads to the activation of pro-osteoclastic genes (e.g., *NFATc1, cathepsin K, TRAP*), osteoclast differentiation (osteoclastogenesis) and then, bone resorption (180, 181). OPG is secreted by stromal and osteoblastic cells acting as a soluble decoy receptor for RANKL, leading to the inhibition of osteoclastogenesis and bone loss (180, 181).

In the OS context, the fact that RANK usually is not expressed by osteoblastic cells and the recognition of RANK^+^ OSCs have been long debated. However, human data and most of established OSC lines confirm the expression of RANK by OSC, proposing the interaction of RANK with its ligand (RANKL) as a critical contributor in OS pathogenesis (181). In this line, a preclinical genetic model of aggressive OS in a RANKL invalidated mice showed the complete blocking of OS development, confirming the critical role of RANK/RANKL signaling into OS progression (182). Moreover, human data analyzing OS tissues from patients with or without metastatic status showed that RANK is expressed in both groups, meaning that RANK expression is not related with the metastatic status, becoming a potential predisposing factor. However, the overexpression of RANKL and the lower OPG/RANK ratio in tumors from metastatic patients lead to hypothesize that the RANKL available in the OS niche is a significant driver to tumor progression and metastasis in OS patients (181). Taken these findings together, strongly support the use of RANKL blockers as a therapeutic approach for OS progression to a metastatic status (181).

On the other hand, the RANKL/RANK/OPG triad has been associated with the pathogenesis of OS by regulating both the osteoclastic activity and, immunoregulatory effects (180, 181). However, the real contribution of osteoclasts to the pathogenesis of OS remains still controversial. Some authors propose that the RANKL-activated osteoclasts might exert a pro-tumoral function in the early stage and on the contrary, a pro-bone remodeling/anti-tumoral effect in the later stage of OS (180, 181). Moreover, osteoclasts may modulate immune response promoting an immunogenic CD4^+^ T cell response upon inflammation. Taken together, osteoclasts can be considered as essential regulators of OS growth and progression through either their resorptive or their immune functions (180, 181).

### Bone Sarcomas and Immune-Based Therapeutic Approaches

In contrast to CS for which the conventional therapy is based on surgery with adequate margins, current treatments of ES and OS associate chemotherapy and surgery. Chemotherapy lines combined a minimum of three cytotoxic agents among doxorubicin, cisplatin, methotrexate, and ifosfamide (168). Unfortunately, most conventional therapies used results in limited therapeutic responses, and new approaches are urgently needed explaining the high number of clinical trials with new drugs for rare cancers including immune modulators, check-point inhibitors and tyrosine-kinase inhibitors (168, 174, 183, 184). Among immune regulators, an activator of macrophages such as muramyl tripeptide phosphatidylethanolamine (MTPPE) showed therapeutic efficacy in the metastatic OS. Trabectedin, a cytotoxic agent, could be attractive to treat sarcomas thanks its effect on macrophage differentiation toward M1 subtypes and targeting of PD1/PDL1 may be promising therapy by disrupting the communications between cancer cells and immune protagonists (183, 184). However, future therapeutic development will require a better characterization of the critical molecular network involved in the differentiation of BS cells and their microenvironment that should lead to the identification of new therapeutic targets and will allow better stratification of the patients enrolled in clinical trials (183, 184).

## Osteoarthritis of the Temporomandibular Joint

### Definition and Pathogenesis of the Osteoarthritis of the Temporomandibular Joint

Degenerative joint disease (DJD) is characterized by the progressive breakdown of articular cartilage, variable degrees of synovial inflammation, and pathological remodeling of subchondral bone (185, 186). Osteoarthritis (OA) is considered the most common form of DJD, affecting approximately 15% of the world population, and a leading cause of pain and disability (187, 188). Although, it mainly affects load-bearing synovial joints, such as the knee, hip, spine, and finger, other joints such as the shoulder or temporomandibular joint (TMJ) could also be affected (189). The TMJ is an exceptional synovial joint that connects the jawbone to the skull and, compared to the knee joint, it is exposed only to limited load-bearing forces (189). It has different morphological, functional, biomechanical, and biological features in comparison to other synovial joints (190). In the knee joint, hyaline cartilage covers articular surfaces, while in the TMJ, the articular lining is covered by fibrocartilage, which is surrounded by an angiogenic microenvironment and is softer than hyaline cartilage (186, 191). Thus, TMJ osteoarthritis (TMJ-OA) should not be considered as a common joint disease, but rather a unique one.

Although different factors such as systemic illnesses, developmental abnormalities, disc displacement, micro-trauma, and parafunction have been associated with the etiology of TMJ-OA, functional overload has been described as its main etiologic factor (28, 189, 192, 193). Articular remodeling is an essential biological process that responds to normal functional loading and ensures the joint's homeostasis (189). However, excessive or unbalanced mechanical loading in the TMJ can induce dysfunctional articular remodeling, leading to degenerative changes (189). Two kinds of mechanical loading occur in the TMJ: Static loading, which occurs during teeth clenching, jaw bracing, and swallowing; and dynamic loading, which occurs during tooth grinding, jaw thrusting, talking, and chewing (194). For instance, the static loading applied during forced mouth opening for 1 day increases the expression of Dickkopf factor-3 (Dkkf-3), an antagonistic non-canonical member of the Wnt family, in the cartilage surface and induces the synthesis of type II and type X collagen in the inner fibrocartilage layers; thus, promoting anabolic effects over the mineralized and unmineralized condylar cartilage (195). Nevertheless, when the same force was applied for 1 week, catabolic effects and several degenerative lesions were observed in the TMJs (196, 197).

Similarly, *in vitro* experiments demonstrated that the effects of loading forces are time-dependent. After 24 h of dynamic compressive loading over condylar chondrocytes, the expression levels of aggrecan, type I and type II collagen increased, possibly as an adaptation attempt; though, after 48 h these expression levels decreased significantly, showing a catabolic effect of prolonged loading (198). Furthermore, compressive forces also promote osteoclastogenesis through the increased expression of RANKL in synovial cells (199). In brief, light forces induced with a mouth opening protocol demonstrated an anabolic effect over TMJ, while massive forces induced a catabolic effect over joint tissues (200). Dynamic overloading forces in a TMJ-OA mice model disrupted the metabolism of hyaluronan (HA), one of the central extracellular matrix (ECM) glycosaminoglycans (GAGs) of the TMJ fibrocartilage (200). In this study, the sustained loading forces significantly decreased the expression levels of hyaluronan synthase (HAS) 2 and 3 and increased the expression levels of hyaluronidase (HYAL) 2 and KIAA1199, an HA binding protein that facilitates the degradation of HA in articular cartilage (200). Interestingly, low-molecular-weight fragments of HA (LMW-HA) can act as a damage-associated molecular pattern (DAMPs), activating antigen presenting cells and initiating the immuno-inflammatory response (201, 202).

### Cellular and the Molecular Immune Basis of the Osteoarthritis of the Temporomandibular Joint

Many contributing factors have been described in the progression of bone changes during TMJ-OA, including genetic factors, female hormones, catabolic enzymes, and inflammatory mediators (203). Although inflammation has considerable importance in the progression of TMJ-OA, it is classified as a “low-inflammatory arthritic condition” as opposed to rheumatoid arthritis (RA), which is considered as a “high-inflammatory condition” (204). Recent studies suggest that OA is an inflammatory disease, at least in certain patients, and that synovial inflammation is accompanied by immune cells infiltration, similarly to RA (205–207). Of these immune cells, macrophages and T lymphocytes are the most abundant cell types that infiltrate the synovia during TMJ-OA, representing approximately 65% and 22% of the total immune cells, respectively (208). Furthermore, several inflammatory cytokines and mediators are increased in the synovial fluid of TMJ-OA affected patients, such as IL-1β, IL-6, IL-17, IFN-γ, TNF-α, prostaglandin E2 (PGE2), and chemerin, suggesting a role of the immuno-inflammatory response during the pathogenesis of the TMJ-OA (209–213). In addition to metabolic or mechanical factors, chronic inflammation induces early damage of the cartilage and consequently initiates biomechanical changes in hard and soft tissues of the joint (214). Thus, the low-grade inflammation present in OA is a result of the interactions between the immune response and local factors, such as tissue breakdown and metabolic dysfunction (215).

The inflammatory response to trauma, hypoxia-reperfusion injury, or chemical-provoked wound typically occurs in the absence of microorganisms; therefore, it has been called “sterile inflammation” (216). The first step of sterile inflammation requires the presence of endogenous molecules released during tissue or cellular injury, which can act as DAMPs able to trigger the immuno-inflammatory response (216). DAMPs can be molecules derived from necrotic cell death, such as high-mobility group box 1 (HMGB1), heat shock proteins (HSPs), or purine-derived metabolites (e.g., ATP); or fragments of molecules derived from the breakdown of the ECM, such as fragments of heparan sulfate, byglican, or HA (e.g., LMW-HA) (215). At initial stages of TMJ-OA, increased local oxidative stress induces the fragmentation of HA in the synovial fluid and fibrocartilage (217–219). The oxidative stress could increase due to direct mechanical trauma, by homolytic fission, or due to hypoxia-reperfusion, and the following non-enzymatic release of reactive oxygen species (ROS) (217, 218). The molecular weight of HA decreases and LMW-HA accumulates within the joint milieu as the disease progresses leading to an increase of joint friction due to the reduction of the chondroprotective and boundary lubrication originally provided by HA (219, 220). Furthermore, LMW-HA can trigger the immune response by interacting with the toll-like receptor (TLR)-2 or TLR-4 expressed in antigen presenting cells (221). TLR activation during OA has been associated with the development of synovitis, cartilage degeneration, and disease susceptibility (222). Using an animal model of TMJ-OA, Kong et al. demonstrated that synovial inflammation changes are related to increased TLR-4 activation and enhanced IL-1β production (223). This synovial inflammatory reaction characterized by the increased levels of IL-1β induced by TLR-4 stimulation depends on the phosphorylation of p38 during the mitogen-activated protein kinase (MAPK) signaling cascade and culminates in the activation of nuclear factor-κB (NF-κB) or nuclear transcription factor activation protein-1 (AP-1) (224). Synovitis is frequently observed during the progression of TMJ-OA (225, 226). The lining layer of synovium is mainly composed of fibroblast-like cells and macrophage-like cells (227). These resident cells play a vital role in the immuno-inflammatory response and bone metabolism during OA by producing several inflammatory mediators that enhance the breakdown of joint tissues (228, 229). Synovial fibroblasts (SF) have the ability to transduce IL-17 signals by expressing different variants of the IL-17 receptor (IL-17R) (227). In response to IL-17A, SFs of the TMJ up-regulate the expression levels of the chemokines CXCL1, IL-8, and CCL20, a specific chemoattractant of Th17 lymphocytes (227). IL-17A also induces increased production of IL-6 by SFs of the TMJ (227), and IL-6 favors the Th17 cell differentiation and promotes osteoclastogenesis and bone resorption (230, 231). Thus, the increased levels of Th17-related chemokines and cytokines produced by SFs of the TMJ stimulated by IL-17A could be related to bone loss and OA progression (227).

In other synovial joints, RANKL is highly expressed on SFs (232), while in TMJ synovium, RANKL is detected in the cytoplasm of synovial lining cells, endothelial cells, and SFs (233). A recent study using Tnfsf11flox/Δ Lck-Cre mice, which lack RANKL expression in T lymphocytes, demonstrated that the absence of RANKL-producing T cells does not protect against osteoclastogenesis and bone resorption (234). On the other hand, the deletion of RANKL on SFs using Tnfsf11flox/Δ Col6a1-Cre mice was protective against osteoclastogenesis and bone loss, thus demonstrating that SFs are the primary RANKL-expressing cells, and responsible for the osteoclast formation and bone resorption during joint inflammation (234). Interestingly, TMJ chondrocytes affected by chondral degradation, may also promote osteoclastogenesis by increasing the RANKL:OPG ratio, ultimately resulting in a subchondral bone loss (235).

LMW-HA is a potent activator of APCs, in particular, dendritic cells (DCs), through the interaction with the complex TLR-4/Cluster of differentiation (CD)44/Myeloid differentiation protein (MD)-2 (236, 237). LMW-HA induces an immuno-phenotypic maturation of DCs through the up-regulation of CD44, CD83, CD80/86, intercellular adhesion molecule-1 (ICAM-1), and the major histocompatibility complex (MHC) II (238). Furthermore, DCs exposed to LMW-HA increase their capacity to stimulate alloreactive T lymphocytes to secrete IL-12, IL-1β, and TNF-α (238). Apart from that, LMW-HA could act as a co-stimulatory molecule during antigen presentation by interacting with CD44 and could also promote the activation and polarization of T lymphocytes (239). The stimulatory effects of LMW-HA over DCs are mediated by the TLR-4 complex signaling pathway, including the phosphorylation of p38 and p42/p44 MAPK and the consequent nuclear translocation of NF-κB (237). LMW-HA also increases the migratory capacity of DCs and stimulates its trafficking toward the draining lymph nodes (240). Moreover, LMW-HA can induce the polarization of DCs to conventional type 1 (cDC1) and type 2 (cDC2) subsets, by increasing the expression levels of their specific transcription factors interferon regulatory factor 4 (IRF4), neurogenic locus notch homolog protein 2 (NOTCH2), and basic leucine zipper ATF-like transcription factor 3 (BATF3) (241). Thus, cDC1 produces TNF-α and cDC2 produces IL-6 and IL-23, inducing the selective differentiation and activation of Th1 and Th17 lymphocytes (242, 243).

Peripheral Th lymphocytes are involved in the pathogenesis of OA (244). T lymphocytes from OA patients can recognize peptides presented by APCs such as the amino acid regions 16–39 and 263–282 located in the G1 domain of human cartilage proteoglycan aggrecan (PG) (245). The recognition of these PG epitopes enhances the proliferation of OA-derived T lymphocytes and increases the production of cytokines, IL-1β, IL-6, IFN-γ, and TNF-α, and CC-chemokines, CCL-2, and CCL-3 (245). Increased expression of the Th1/Th17/Th22 cytokines IL-1β, IL-17, and IL-22, chemokines CCL5, and CCL20, and chemokines receptors CCR5 and CCR7 have been detected in synovial cells of TMJ-OA affected patients (212, 246). Further, the increased levels of IL-1β, IL-17, and IL-22 significantly correlate with the enhanced RANKL expression and immunological signs of bone degeneration (246). Moreover, the synovial fluid obtained from TMJ-OA affected patients induces significantly more osteoclast maturation and activity in comparison to synovial fluid obtained from controls (246).

Although both Th1 and Th17 cells are involved in the etiology of OA, it has been reported that the IL-23/IL-17 axis is more critical than the IL-12/IFN-γ axis in the onset of the disease (247–249). Analyses of blood samples obtained from OA affected patients, and healthy donors showed a significantly higher percentage of activated CD4+ T cells and Th17 lymphocytes in the OA group, while there were no differences between the percentages of Th1 and Th2 lymphocytes among the studied groups (250). Further, increased numbers of Th17 cells have also been detected in the OA synovial membrane (251). An osteoarthritic joint milieu with low levels of TGF-β but high levels of IL-12 induces the plasticity of Th17 lymphocytes to an intermediate phenotype between Th1 and Th17 lymphocytes known as Th17/1 cells. These cells are characterized by an increased expression of the transcription factors retinoic acid-related orphan receptor C2 (RORC2), and T-box expressed in T cells (T-bet) and production of the cytokines IFN-γ and IL-17 (252). Indeed, in OA patients, both peripheral blood and synovial fluid frequencies of Th17/1 cells are significantly increased in comparison to healthy subjects or even rheumatoid arthritis patients (253). Additionally, the enrichment of Th17/1 cells in OA patients is higher in synovial fluid than serum (253). These findings suggest that Th17/1 cells could be a Th subset with a particular role during OA and; thus, need to be further evaluated in TMJ-OA.

Higher expression levels of IL-17 have been reported in either synovial membranes or synovial fluid of TMJ-OA affected patients (212, 246, 254, 255). IL-6, a key cytokine involved in Th17 polarization, is also increased in the TMJ-OA affected patients (256). *In vitro* experiments have demonstrated that IL-17A promotes synovial hyperplasia, synoviocyte invasion, cartilage breakdown, and angiogenesis (257–260). Using SFs isolated from patients with TMJ disorders, Hattori et al. determined that IL-17A upregulates the expression of IL-6, CXCL1, IL-8, and CCL20, in a dose- and time-dependent manner, promoting T lymphocyte chemoattraction toward the TMJ synovial tissues (227). However, the central role of the Th17 lymphocytes during the pathogenesis of the joint disorders is related to its RANKL-producing osteoclastogenic function (261).

### Impact of Osteoimmunology in the Osteoarthritis of the Temporomandibular Joint

Different types of cells orchestrate the physiological remodeling process within the TMJ bone microenvironment. As mentioned before, osteoclasts and osteoblasts are the primary effector cells involved in the bone resorption and formation, respectively, in the articular subchondral bone. In this molecular-and-cellular-regulated process, however, the contribution of other cell types, such as osteocytes, has been considered. Osteocytes are a group of cells which differentiate from osteoblasts, that during the formation of mineralized tissues are left embedded within the bone matrix, and further contribute to the regulation of bone metabolism (262). Even though, under physiological conditions, osteoblasts are considered as the primary source of RANKL for the RANKL-induced osteoclastogenesis and consequent osteoclast-mediated subchondral bone remodeling; osteocyte-specific RANKL-deficient mice (*Tnfsf11*^flox/Δ^ *Dmp1-Cre* or *Tnfsf11*^flox/flox^ *Sost-Cre* mice) present a similar osteopetrotic phenotype than RANKL-null mice; thus, demonstrating the importance of osteocytes as a primary source of RANKL (5, 263). Moreover, by using a high-purity isolation method for osteocytes and osteoblasts, Nakashima et al. evidenced that osteocytes have a stronger ability to induce and support osteoclastogenesis than osteoblasts, through higher *Tnfsf11* (encoding RANKL) mRNA expression and RANKL production (5). Apart from that, the authors demonstrated that the absence of RANKL in T cells is not critical for bone metabolism in physiological conditions (5).

Osteocytes also actively release RANKL in response to mechanical stress (5). Indeed, osteocytes contact osteoclast precursor cells and mature osteoclasts through long dendrites that reach the bone surface, which enable direct cell-cell interaction by membrane-expressed factors released in response to load forces, such as RANKL (264). A mechanical stress experiment with MLO-Y4 osteocyte-like cells revealed that *Tnfsf11* expression is remarkably induced by mechanical strength (5). In fact, during the application of orthodontic forces for bone remodeling-dependent tooth movement, osteocytes were the primary source of RANKL in response to compressive forces, thus promoting osteoclastogenesis and bone resorption (265). Conversely, increased loading forces during mastication induced by a hard diet in mice showed an increase in the osteocyte-mediated bone formation, through an increment of the levels of insulin-like growth factor (IGF)-1 and thus, promoting osteoblastogenesis (266). Therefore, the osteocyte response to mechanical load could drastically differ between the physiological context and the pathological scenario during TMJ-OA. Indeed, osteocytes adjacent to sites of bone microdamage, as occurs in subchondral bone during the advanced stage of TMJ-OA, undergo apoptosis; whereas osteocytes adjacent to this apoptotic cells upregulate the expression of osteoclastogenic and immunogenic signaling molecules, such as ATP, membrane-derived peptides, chemokines, vascular endothelial cell growth factor A (VEGFA), and RANKL (262). Besides, osteocytes exposed to extra-cellular matrix molecules derived from TMJ-OA subchondral bone osteoblasts showed decreased levels of maturation and increased levels of apoptosis due to a decrease in the integrin-β1 expression, in comparison with extra-cellular matrix molecules derived from normal subchondral bone osteoblasts (267). This suggests that the pathological behavior of osteocytes in response to the mechanical overload during the progression of TMJ-OA could be due to molecular and cellular changes occurring in the joint microenvironment.

**Figure 4:**
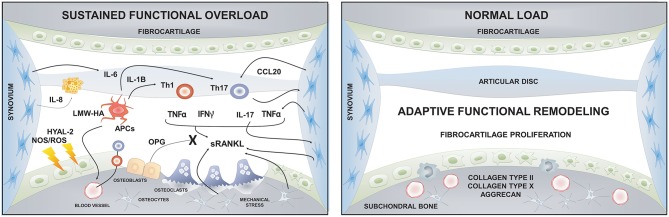
Immune response and bone crosstalk during temporomandibular joint osteoarthritis. The sustained functional overload that exceeds the joint adaptive capacity can induce dysfunctional remodeling. This dysfunctional remodeling is characterized by increased oxidative stress, due to hypoxia/reperfusion effect, and higher levels of catabolic enzymes that degrade the fibrocartilage extracellular matrix and induce chondrocyte apoptosis. The molecular products derived from cartilage breakdown (e.g., LMW-HA or PG) can trigger the immuno-inflammatory response by interacting with APCs and finally resulting in the activation of Th17 lymphocytes and the RANK/RANKL/OPG axis, leading to subchondral bone resorption. The normal joint load of the TMJ, such as static loading during swallowing or teeth clenching, induces an adaptive functional remodeling of joint tissues and promotes fibrocartilage healing. HYAL, hyaluronidase; LMW-HA, low molecular weight hyaluronan; NOS, nitric oxide; OPG, osteoprotegerin; ROS, reactive oxygen species; sRANKL; soluble receptor activator of nuclear factor-kappa B ligand; APCs, Antigen presenting cells.

The RANKL:OPG ratio is increased in synovial fluid obtained from TMJ-OA patients, mainly due to the increased levels of RANKL together with the decreased levels of OPG detected in the joint microenvironment (212, 268). The secretion of IL-17 by Th17 lymphocytes enhances the RANKL production by osteoblasts, osteocytes, and SFs and activates the production of other osteoclastogenic cytokines, such as TNF-α, IL-1β, and IL-6 by synovial macrophages (269). Thus, the accumulation of Th17 lymphocytes in synovial tissues may contribute to subchondral bone resorption by stimulating the RANKL-mediated osteoclast activity (270). Kikuta et al. using intravital multiphoton microscopy, have demonstrated that Th17 but not Th1 lymphocytes preferentially adhere to mature osteoclasts and that low levels of RANKL secreted by mature osteoclast-adhered Th17 lymphocytes could induce the rapid conversion from moving-non-resorptive to static-bone-resorptive osteoclast phenotype in bone (271). Besides, using a mice model of primary hyperparathyroidism, it was described that the osteocyte-mediated RANKL production induced by Th17-derived IL17A/IL17RA interaction is critical for the bone catabolic activity, revealing another potential mechanism of subchondral bone loss induction by Th17 cells during TMJ-OA (74). Altogether, these studies demonstrated a pivotal role for Th17 lymphocytes in the osteoimmunology of the TMJ-OA, through the modulation of the osteoblast/osteocyte/osteoclast activity (Figure 4).

Overall, the evidence presented above shows that TMJ-OA could be considered a chronic mechanically induced and immuno-inflammatory-mediated disease, mainly due to the continuous production of DAMPs during joint tissues destruction, particularly LMW-HA, the activation of a Th17-pattern of immune response and the consequent RANKL-mediated and osteoclast-induced bone resorption.

### Translational Applications

For the diagnosis of the TMJ-OA, four criteria are usually used: Joint noises, chronic joint pain, joint cramping during movements of the jaw, and degenerative bone deterioration detected through imaging (111, 214, 272). However, these criteria are only observable when the disease has been established, and tissue damage has occurred; so these gold-standard criteria do not allow early symptoms detection to prevent or stop the progression of TMJ-OA. In addition, when the diagnosis has been made, the analysis of treatment success is based on exactly the same criteria and, in older adults, these criteria are unreliable, due to the subjective component of clinical symptoms and the involuntary movements that many individuals present, which diminish the sharpness of the images and in some cases making their contribution, as a complement to the diagnosis, uncertain (273).

Several research groups have proposed to incorporate competing molecular strategies that could allow the early diagnosis of TMJ-OA and the evaluation of its therapeutic success (256, 273–281). In this sense, the determination of molecular mediators associated with the inflammatory and destructive articular tissue processes characteristic of TMJ-OA is convenient; however, nowadays, there are no registered initiatives focused on the development of an alternative solution complementary to the current standards for the diagnosis of the disease.

In this sense, the validation of a diagnostic strategy based on the identification of a panel of detectable biomarkers in the synovial fluid of the TMJ and complementary to the traditional clinical-imaging methods of diagnosis of TMJ-OA is feasible. An ideal diagnostic panel should incorporate osteoimmunology markers. In this way, the identification of molecular mediators associated with the differentiation and activity of osteoblasts, osteoclasts and/or osteocytes, as well as cytokines (IL-1β, IL-6, IL-17, IL-12, and TNF-α), MMPs (MMP-8 and MMP-9), and RANKL, could be proposed as potentially sensitive and specific biomarkers to detect early degenerative changes in the TMJ.

## Conclusions

The understanding of the immune-mediated bone destruction during periodontal diseases, periapical infections, maxillary bone-sarcomas, and temporomandibular joint osteoarthritis require a detailed analysis of a wide array of pathways. While each disease in the oral and maxillofacial milieu has a distinct etiological basis, recent work has demonstrated that several mechanistic aspects could share common cellular and molecular processes that are unique to the oral and maxillofacial structures. In this work, we have reviewed the cutting-edge literature in an attempt to identify the most current knowledge in the oral osteoimmunology to provide new therapeutic approaches in otherwise difficult to treat bone lesions. The characterization of the different molecules involved in immune-mediated tissue destruction has been identified to provide biomarkers that would be useful to comprehend the link between bone-destructive oral diseases, such as periodontal disease and apical periodontitis, with systemic diseases. Collectively, the work represents a unique attempt to tackle common pathways of osteoimmunology and osteoinflammation of the oral cavity, which presents a highly unique environment colonized by the highest number of bacterial species in the mammalian body and regulated by highly functional biomechanical forces created by occlusion. Thus, successful prevention and treatment of oral diseases require recognition of this complexity to design specialized therapeutic approaches and maintain the treatment outcomes.

## Author Contributions

CA, RV, and AK conceived the original idea and designed the manuscript. TS, PP, H-ML, LG, and MH provided the data. CA, FC, LC, GM, and MH designed the figures. GG edited the figures. All authors participated in manuscript writing and critically reviewed the manuscript. AK edited the manuscript and supervised the project.

### Conflict of Interest Statement

TS is an inventor of US-patent 5652223, 5736341, 5866432, 6143476, 20170023571/A1, and patent 127416. LG and H-ML are listed on patents on the medications/compounds described in this paper, which have been fully assigned to their institution, Stony Brook University, State University of New York and financially supported by NIDCR/NIH, R37-DE03987, K16DE-00275, K11DE00363, R01DE012872, 1R41DE024946, R42-DE024964, and additional support from U.S. Dept. of Defense (DoD); Johnson & Johnson, Collagenex Pharma., Inc., Galderma R&D, Kroc Foundation for Medical Res., Traverse Biosciences, Inc., N.Y. State Diabetes Assoc., Stony Brook University Center for Advanced Biotechnology. The remaining authors declare that the research was conducted in the absence of any commercial or financial relationships that could be construed as a potential conflict of interest.
